# Sociodemographic factors associated with the use of mental health services in depressed adults: results from the Korea National Health and Nutrition Examination Survey (KNHANES)

**DOI:** 10.1186/s12913-014-0645-7

**Published:** 2014-12-20

**Authors:** Se Jin Park, Hong Jin Jeon, Ju Young Kim, Sohye Kim, Sungwon Roh

**Affiliations:** Department of Mental Health Research, Seoul National Hospital, Seoul, 143-711 Korea; Depression Center, Department of Psychiatry, Samsung Medical Center, Sungkyunkwan University School of Medicine, Seoul, 135-710 Korea; Depression Clinical and Research Program, Department of Psychiatry, Massachusetts General Hospital, Harvard Medical School, Boston, MA 02114 USA; Department of Family Medicine, Seoul National University Bundang Hospital, Seongnam-si, Gyeonggi-do 463-707 Korea; Department of Medical Nutrition, Graduate School of East–west Medical Science, Kyung Hee University, Yongin-si, Gyeonggi-do 446-701 Korea; Center for Addiction Medicine, Department of Psychiatry, Massachusetts General Hospital, Harvard Medical School, Boston, MA 02114 USA

**Keywords:** Mental health service, Use, Depressive mood, Sociodemographic factor, Age, Education

## Abstract

**Background:**

The aims of this study were to determine the utilization of mental health services (MHSs) by adults with a depressive mood and to identify the influencing sociodemographic factors, using a nationwide representative Korean sample.

**Methods:**

The study included 2735 subjects, aged 19 years or older, who had experienced a depressive mood continuously for over 2 weeks within the previous year, using the data from the KNHANES IV (Fourth Korea National Health and Nutrition Examination Survey), which was performed between 2007 and 2009, and involved a nationally representative sample of the Korean community population who were visited at home. A multivariate logistic regression analysis was used to estimate the adjusted odd ratios (ORs) and 95% confidence intervals (CIs) for the use of MHSs, which was defined as using healthcare institutions, consulting services, and inpatient or outpatient treatments due to mental health problems.

**Results:**

MHSs had been used by 9.6% of the subjects with a depressive mood. The use of the MHSs was significantly associated with age, education level, and employment status, after adjusting for sociodemographic and health-related factors. Specifically, the OR for the nonuse of MHSs by the elderly (≥65 years) relative to subjects aged 19–34 years was 2.55 (95% CI = 1.13–5.76), subjects with a lower education level were less likely to use MHSs compared to those with a higher education level (7–9 years, OR = 2.35, 95% CI = 1.19–4.64; 10–12 years, OR = 1.66, 95% CI = 1.07–2.56; ≥13 years, reference), and the OR of unemployed relative to employed was 0.47 (95% CI = 0.32–0.67).

**Conclusions:**

Among Korean adults with a depressive mood, the elderly, those with a lower education level, and the employed are less likely to use MHSs. These findings suggest that mental health policies should be made based on the characteristics of the population in order to reduce untreated patients with depression. Greater resources and attention to identifying and treating depression in older, less educated, and employed adults are warranted.

## Background

Depression is one of the most common diseases worldwide, and has a heavy socioeconomic burden [1,2]. Depression has been ranked third on the World Health Organization’s list of medical conditions with the greatest disease burden worldwide, and is expected to top that list by 2030. The 1-year prevalence of a major depressive disorder was reportedly 6.6% in the USA [3], 2.9% in Japan [4], and 2.5% in Korea [5]. An epidemiological study in Korea found that major depression had a high disease burden, with a disability-adjusted life years (DALYs) value of 1,287 years (per 100,000 persons), representing 49% of the burden of all mental diseases [6]. Moreover, depression significantly influences health outcomes, such as disability, premature mortality, comorbidity with chronic disease, and decreased quality of life, in both Western countries [7] and Korea [8]. Despite the high prevalence and social burden of depression, only a small percentage of people with depression use psychiatric services [4,9]. Furthermore, the majority of adults with mental disorders, including depression, do not seek help from mental health services (MHSs) [10,11].

In order to provide effective treatment for people suffering from mental health problems, it is critical to identify the barriers that they face when accessing MHSs [12]. Previous studies have revealed that such barriers include structural factors (for example, the cost of services) and attitude factors (for example, negative perception and prejudice against mental disorders) [13,14]. These attitudes toward mental disorders differ according to sociodemographic characteristics such as age, gender, and education level [14,15]. Therefore, the individuals’ sociodemographic characteristics may directly or indirectly influence their use of MHSs [16]. Several previous studies have found that men [16,17], adolescents, and seniors [10,12,16,18] with a low socioeconomic status [12,18,19] or living in rural areas [20] were less likely to access MHSs.

Factors influencing the use of MHSs are various according to studies as mentioned above because each country has a unique healthcare delivery system. The Korean health insurance system is mainly run by the national government as in European countries, but most of health service providers are private hospitals. People pay the insurance dues differently in grade based on their income, and medical services for recipients of livelihood program are free of charge while medical care is equivalent [21].

Many studies have investigated the barriers to the use of MHSs in European Union countries and the USA; however, very little has been uncovered about the factors that affect MHS use for depression in Asian countries, and particularly in Korea.

The aims of this study were to determine the use conditions of MHSs and to identify the sociodemographic factors associated with MHS use after considering the effect of mental health related factors among adults with a depressive mood, using a nationwide representative Korean sample.

## Methods

### Data source and study samples

**Figure 1:**
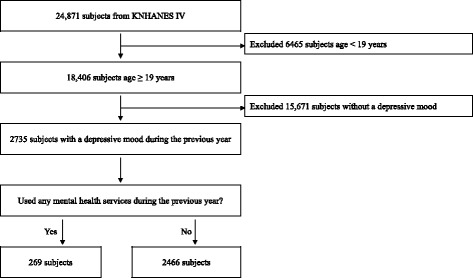
**Flowchart of the study population KNHANES IV (the Fourth Korea National Health and Nutrition Examination Survey)**.

The data used in this study were obtained from the Fourth Korea National Health and Nutrition Examination Survey (KNHANES IV), which was conducted during 2007–2009 by the Korea Centers for Disease Control and Prevention (KCDC). The KNHANES is a nationally representative and reliable study that assessed health status, health behaviors, and nutritional status. The survey used a stratified, multistage, probability-sampling design to represent the entire Korean population. The KNHANES is composed of the Health Interview Survey, the Health Examination Survey, and the Nutrition Survey. The Health Interview Survey was performed using self-administered structured questionnaires to obtain information regarding sociodemographic characteristics, health status, health service use, and health behaviors. Trained interviewers visited each household and assisted the participants with specific items in the self-administered tool. The KNHANES IV surveyed household members aged over 1 year (*n* = 24,871) from a total of 9421 households (response rate 78.4%). All subjects in the survey participated voluntarily with informed consent, and the survey protocol was approved by the Institutional Review Board of the KCDC. This study is in compliance with the Helsinki Declaration, and was exempted from the evaluation of Seoul National Hospital Institutional Review Board in 2014. This study ultimately included 2735 subjects aged ≥19 years that had continuously experienced a depressive mood for more than 2 weeks within the previous year (Figure 1).

### Measurements

Depressive mood was assessed by a “yes” or “no” answer to the question: “Have you felt sadness or despair affecting your daily life for more than 2 weeks over the past year?” [22]. The use of MHSs included subjects who had visited healthcare institutions or had received consulting services by phone or via the Internet for mental health problems. The questions were as follows:

- “Have you visited any healthcare institutions, or have you received consultation through the Internet, telephone, etc. due to your mental health problems during the past year?”
- “Have you experienced inpatient treatment for depression during the past year?”
- “Have you experienced outpatient treatment for depression during the past 2 weeks?”

Sociodemographic factors included gender, age, region (urban or rural), education level (≤6 years, 7–9 years, 10–12 years, or ≥13 years), employment status (employed or unemployed), monthly household income (<US$1000, US$1000 to < US$3500, or ≥ US$3500), national health insurance type (national insurance or medical aid), and marital status (married, never married, divorced, or widowed).

In addition, smoking status (current, past, or never) and alcohol consumption status (current, past, or never) were included, and the subjects’ mental and physical health status (for example, perceived usual stress, subjective health status, and chronic conditions) were also assessed. Perceived usual stress was measured by the question, “How do you usually feel stress in your daily life?”, with responses provided on a 4-point Likert scale (very high, high, low, or little). Subjective health status was measured by the question, “Generally, how is your subjective physical health status?”, with responses provided on a 5-point Likert scale (very poor, poor, fair, good, or very good). Finally, chronic conditions such as arthritis, diabetes, hypertension, angina, and asthma were included. Each disease was organized into clinically diagnosed cases by self-reporting; for example, “Have you been diagnosed with diabetes by a physician?” was categorized into two groups: yes or no.

### Statistical analyses

Given the complex sampling design of the KNHANES IV, weighted values were applied by using the survey-related procedure of SPSS software version 21 in all analyses. For variable selection, we included all sociodemographic variables as well as health-related variables associated with substance use, stress and chronic disease from the survey data.

The general characteristics of the study sample were tabulated. A chi-square test was used to compare the differences in sociodemographic factors, health behaviors, and health-related factors between the two groups, according to the use of MHSs among the subjects with a depressive mood. Univariate and multivariate logistic regression were used to estimate the odds ratios (ORs) and 95% confidence intervals (95% CIs) of MHS use for each measure. Specifically, a multivariate logistic regression model was used to investigate sociodemographic factors associated with the use of MHSs after fully adjusting for all evaluated covariates such as sociodemographic and health-related factors. The level of statistical significance was set at *P* < 0.05.

## Results

### Characteristics of the subjects

Of the 18,406 subjects aged ≥19 years who participated in the KNHANES IV, 2735 (15.8%) had experienced a depressive mood that hindered their daily life during the previous year. Among these subjects, 1953 (66.5%) were women, they were aged 48.00 ± 0.44 years (mean ± SE), and elderly aged ≥65 years accounted for 20.4% of the sample. Of the 2735 subjects who had experienced a depressive mood, only 9.6% had used MHSs, 32.7% had an education level of ≤6 years, 18.8% were divorced or widowed, and 55.2% had the lowest monthly household income (<US$1000). Furthermore, 60.9% usually felt high or very high levels of stress in their daily life, 38.7% perceived that their health status was poor or very poor, and 35.3% had a diagnosis of at least one or more of five chronic diseases (arthritis, diabetes, hypertension, angina, and asthma; Table 1).

**Table 1:** **Characteristics of the study sample (** ***n*** **= 2735; age = 48.00 ± 0.44 years, mean ± SE)**

**Characteristic**	***n***	**%**
**Gender**		
Men	782	33.5
Women	1953	66.5
**Age group, years**		
19–34	459	24.8
35–49	703	29.7
50–64	745	25.1
≥ 65	828	20.4
**Mental health services use**		
Yes	269	9.6
No	2466	90.4
**Residential region**		
Urban	1973	80.1
Rural	762	19.9
**Education level, years**		
≤ 6	1146	32.7
7–9	336	12.0
10–12	773	35.0
≥ 13	467	20.3
**Marital status**		
Married	1793	63.5
Widowed	468	12.9
Divorced	150	5.9
Never married	314	17.7
**Employment status**		
Employed	1348	52.2
Unemployed	1355	47.8
**Monthly household income**		
< US$1000	1612	55.2
US$1000 to < US$3500	566	25.1
≥ US$3500	475	19.7
**National health insurance type**		
National insurance	2469	93.0
Medical aid	264	7.0
**Smoking status**		
Current	554	25.1
Past	412	15.3
Never	1768	59.6
**Alcohol consumption status**		
Current	1025	31.2
Past or never	1708	68.8
**Subjective health status**		
Very good	65	2.7
Good	620	24.3
Fair	836	34.3
Poor	908	30.0
Very poor	294	8.7
**Perceived usual stress**		
Very high	474	17.9
High	1139	43.0
Low	936	33.3
Little	185	5.8
**Ever diagnosed with a chronic disease**		
Arthritis	613	17.2
Diabetes	281	7.9
Hypertension	682	20.3
Angina	111	2.9
Asthma	141	4.5
**Presence of chronic diseases** ^**a**^		
Yes	1183	35.3
No	1552	64.7

Note: The sum of numbers in the subgroups does not equal the total number of subjects in this study; subjects with missing values were excluded.

*n* = unweighted sample size, % = population-weighted proportions, SE = standard error.

^a^With one or more of five chronic diseases: arthritis, diabetes, hypertension, angina, and asthma.

### Use of mental health services according to sociodemographic and health-related factors

Table 2 lists the differences in the use of MHSs according to each sociodemographic or health-related factor among subjects with a depressive mood. Use of MHSs was significantly lower among men (7.4%) compared to women (10.6%). Older subjects, those living rurally and those with a lower level of education appeared less likely to use MHSs, but these findings were not statistically significant. However, the use of MHSs was significantly higher among the unemployed compared to those who were in work (13.0% vs. 6.6%), and among those with a poorer subjective health status (OR = 0.73, 95% CI = 0.63–0.85) and the highest perceived usual stress (OR = 0.50, 95% CI = 0.27–0.91).

**Table 2:** **Sociodemographic and health-related characteristics according to use or nonuse of mental health services among subjects with a depressive mood (age ≥19 years)**

**Variable**	**Mental health services use during the previous year**					
	**Use (** ***n*** **= 269)**		**Nonuse (** ***n*** **= 2466)**		**Unadjusted model** ^**a**^	
	***n***	**%**	***n***	**%**	**OR**	**(95%CI)**
**Sociodemographic factors**						
Gender						
Men	56	7.4	726	92.6	1.48	(1.05-2.10)
Women	213	10.6	1740	89.4	1.00	
Age group, years						
≥ 65	69	8.1	759	91.9	1.35	(0.85-2.13)
50–64	65	8.1	680	91.9	1.34	(0.86-2.10)
35–49	82	11.0	621	89.0	0.96	(0.63-1.48)
19–34	53	10.6	406	89.4	1.00	
Residential region						
Urban	211	9.9	1762	90.1	1.27	(0.87-1.85)
Rural	58	8.0	704	92.0	1.00	
Education level, years						
≤ 6	98	8.9	1048	91.1	1.48	(1.00-2.19)
7–9	29	7.3	307	92.7	1.85	(1.00-3.27)
10–12	85	9.3	688	90.7	1.41	(0.94-2.12)
≥ 13	56	12.6	411	87.4	1.00	
Marital status						
Widowed	39	8.0	429	92.0	1.30	(0.88-1.92)
Divorced	16	10.0	134	90.0	1.06	(0.61-1.87)
Never married	30	8.9	284	91.1	1.16	(0.72-1.87)
Married	184	10.2	1069	89.8	1.00	
Employment status						
Unemployed	175	13.0	1180	87.0	0.48	(0.35-0.64)
Employed	94	6.6	1254	93.4	1.00	
Monthly household income						
< $1000	167	10.8	1445	89.2	0.72	(0.49-1.07)
≥ $3500	48	8.9	427	91.1	0.90	(0.55-1.46)
$1000 to < $3500	48	8.1	518	91.9	1.00	
National health insurance type						
Medical aid	33	12.5	204	87.5	0.73	(0.47-1.14)
National insurance	235	9.4	2234	90.6	1.00	
**Health-related factors**						
Smoking status						
Current	48	7.9	506	92.1	1.33	(0.89-1.98)
Past	36	9.4	376	90.6	1.11	(0.72-1.70)
Never	185	10.3	1583	89.7	1.00	
Alcohol consumption status						
Current	170	10.5	1538	89.5	1.16	(0.84-1.60)
Past or never	99	9.2	926	90.8	1.00	
Subjective health status						
Very good	5	7.7	60	92.3	1.31	(1.09-1.58)
Good	40	6.1	580	93.9		
Fair	73	8.5	763	91.5		
Poor	113	12.7	795	87.3		
Very poor	38	13.6	256	86.4		
Perceived usual stress						
Very high	67	13.2	407	86.8	0.50	(0.27-0.91)
High	117	10.0	1022	90.0	0.72	(0.40-1.27)
Low	71	7.3	865	92.7	1.00	(0.55-1.81)
Little	14	8.5	171	91.4	1.00	
Ever diagnosed with a chronic disease						
Arthritis						
Yes	72	12.6	541	87.4	0.98	(0.49-0.94)
No	197	8.9	1925	91.1	1.00	
Diabetes						
Yes	23	6.4	258	93.6	1.59	(0.96-2.64)
No	246	9.8	2208	90.2	1.00	
Hypertension						
Yes	66	9.1	616	90.9	1.08	(0.76-1.52)
No	203	9.7	1850	90.3	1.00	
Angina						
Yes	10	9.1	101	90.9	1.06	(0.48-2.33)
No	259	9.6	2365	90.4	1.00	
Asthma						
Yes	20	16.2	121	83.8	0.53	(0.29-0.95)
No	249	9.2	2345	90.8	1.00	
Presence of chronic diseases^b^	120	10.5	1063	89.5	0.85	(0.63-1.15)
	149	9.1	1403	90.9	1.00	

^a^Subjective health status was performed as continuous variable.

^b^With one or more of five chronic diseases: arthritis, diabetes, hypertension, angina, and asthma.

### Association between use of mental health services and sociodemographic factors

The results of the multivariate logistic regression analyses are presented in Table 3. In the adjusted model, the OR for the use of MHSs by the elderly (≥65 years) relative to subjects aged 19–34 years was 2.55 (95% CI = 1.13–5.76), but the difference was not found to be significant in the unadjusted model (Table 2). Moreover, subjects with a lower education level were less likely to use MHSs compared to those with a higher education level (7–9 years, OR = 2.35, 95% CI = 1.19–4.64; 10–12 years, OR = 1.66, 95% CI = 1.07–2.56; ≥13 years, reference). Conversely, the OR for the unemployed group relative to the employed group was 0.47 (95% CI = 0.32–0.67).

**Table 3:** **Sociodemographic and health-related characteristics associated with nonuse of mental health services among subjects with a depressive mood (age ≥ 19 years)**

**Variable**	**Adjusted model** ^**a**^	
	**OR**	**(95% CI)**
**Sociodemographic factors**		
Gender		
Men	1.07	(0.66–1.75)
Women	1.00	
Age group, years		
≥ 65	2.55	(1.13-5.76)
50–64	1.63	(0.82-3.24)
35–49	1.04	(0.59-1.84)
19–34	1.00	
Residential region		
Urban	1.08	(0.72–1.62)
Rural	1.00	
Education level, years		
≤ 6	1.87	(0.97-3.60)
7–9	2.35	(1.19-4.64)
10–12	1.66	(1.07-2.56)
≥ 13	1.00	
Marital status		
Widowed	1.40	(0.84–2.34)
Divorced	1.29	(0.67–2.45)
Never married	1.39	(0.74–2.62)
Married	1.00	
Employment status		
Unemployed	0.47	(0.32–0.67)
Employed	1.00	
Monthly household income		
< US$1000	0.65	(0.41–1.03)
≥ US$3500	0.89	(0.54–1.46)
US$1000 to < US$3500	1.00	
National health insurance type		
Medical aid	0.82	(0.50–1.36)
National insurance	1.00	
**Health-related factors**		
Smoking status		
Current	1.11	(0.66-1.88)
Past	1.00	(0.57-1.66)
Never	1.00	
Alcohol consumption status		
Current	1.10	(0.76-1.61)
Past or never	1.00	
Subjective health status	0.71	(0.60-0.85)
Perceived usual stress		
Very high	0.94	(0.44-1.99)
High	1.19	(0.58-2.45)
Low	1.45	(0.70-3.01)
Little	1/.00	
Ever diagnosed with a chronic disease		
Arthritis		
Yes	0.68	(0.45-1.02)
No	1.00	
Diabetes		
Yes	1.77	(1.02-3.08)
No	1.00	
Hypertension		
Yes	1.02	(0.67-1.56)
No	1.00	
Angina		
Yes	1.04	(0.44-2.48)
No	1.00	
Asthma		
Yes	0.65	(0.35-1.21)
No	1.00	
Intercept (coefficient, CI)	2.9133	(1.8018-4.0248)

OR = odds ratio, CI = confidence interval, if OR > 1 then less use of mental health services, and OR < 1 then more use of mental health services.

^a^Adjusted model: adjusted for sociodemographic and health-related factors (smoking status, alcohol consumption status, usual stress awareness, subjective health status, and ever diagnosed with a chronic disease such as arthritis, diabetes, hypertension, angina, or asthma).

There was a significant affect of gender in the unadjusted model (Table 2), in that men were less likely to use MHSs; however, this result was not statistically significant after adjusting for all factors. Finally, being elderly (≥65 years), in the lower education group, and employed was strongly associated with a lower use of MHSs.

## Discussion

People worldwide suffering from psychiatric diseases including depression exhibit a low rate of MHS use, as shown in the present study, in which only 9.8% of adults who experienced depressive moods for more than 2 weeks over the previous year had used MHSs. In the Epidemiological Survey of Mental Disorders in Korea, the prevalence of MHS use was 15.3% among people who had one or more psychiatric disease [5]. In the USA the prevalence was 13% for those reported with a depressive mood [23], 57.3% for major depression [3], and 19% for a substance use disorder [10], demonstrating a low treatment rate among psychiatric patients. However, Korean adults with psychiatric problems demonstrated a far lower usage rate than their counterparts in the USA, which suggests that the obstacles to MHSs accessibility are more serious in Korea than in the USA. Obstacles to the use of MHSs include lack of awareness of the necessity of MHSs [10], patients’ attitudes regarding self-treatment, low recognition of their diseases, belief in natural recovery, negative perception and prejudice against the use of MHSs, and economic burden [11].

Using a nationwide representative Korean sample, the present study demonstrated an association between sociodemographic factors and MHS use in subjects aged over 19 years who had experienced a depressive mood. According to Andersen’s model, the use of healthcare services is affected compositely by predisposing factors (gender, age, education, marital status, employment status, occupation, and attitude) and promoting factors (income, health insurance, and geographical accessibility) [24]. Variations in the sociodemographic characteristics of individuals create differences in the use of MHSs [16]. Furthermore, the severity of psychiatric disease is considered an important factor in MHS use [12,25-27]. That is, the rate of MHS use increases with the disease severity. It is therefore important to consider the disease severity in order to clearly evaluate the effects of sociodemographic characteristics on service use [12,28]. In this sense, a major limitation of the present study was that the severity of the depressive mood could not be evaluated.

The present findings show that after fully adjusting for all evaluated factors such as sociodemographic and health-related factors, age, education level, and employment status significantly influenced the use of MHSs. Previous studies have found that MHS use differs according to gender; specifically, that women use MHSs more than men [16,25]. However, in the present study, service use by women was only higher than that of their male counterparts in the unadjusted analysis. Regarding this gender difference, it has been acknowledged that women are more open about their psychiatric problems, and generally have a more positive attitude toward mental diseases [29]. In particular, there are fewer stigmas associated with depression among women than men [14]. Therefore, women are more likely to recognize the necessity of MHSs [10,17,18]. Prejudice and stigma toward MHSs are strongly correlated with actual service use [15]. The lower prejudice and more positive attitudes among women in this regard may explain their high MHS use. However, some researchers argue that the gender difference is mainly attributable to the exposure to depression being greater for women than for men [30], and that once socioeconomic variables are adjusted, the difference reduces or disappears [11]. Similarly, although in the present sample there were more women with a depressive mood than men, the gender difference disappeared after adjusting for sociodemographic factors.

### Differences in MHS use between the age group

Regarding the difference in MHS use between the age groups, some previous studies have produced varying results among the adolescent, middle-aged, and elderly [10,11,16,18,20,31,32], while others have found that age was not associated with MHS use [27]. However, those aged over 65 years in the present study were less likely to use services than their younger counterparts. According to previous reports, the elderly are less sensitive to psychiatric symptoms and confuse such symptoms with those of the natural aging process, thus preferring treatment at general medical centers rather than at specialized MHS institutions [20]. In contrast, younger people are more aware of the necessity of MHSs, resulting in middle-aged people to use services more frequently [10]. An exception to this pattern was found in a study conducted in Iceland, in which the elderly were found to have visited more mental health institutions and sought help from psychiatrists more frequently. However, these results were explained by favorable conditions in Iceland, namely an increase in free time and a low-cost health insurance system available to those aged over 67 years [16]. On the other hand, while stigma against depression varies little with age, the effect of the stigma associated with mental illness has a stronger impact on certain age groups [14], and particularly among the elderly with depression, stigma is a significant obstacle to their use of MHSs [31]. The rapidly expanding aged population and depression-related suicide among the aged have recently emerged as growing social problems in Korea [33]. To effectively deal with these problems, greater public health strategies such as education, counseling, and campaigning for older people are required to promote their accessibility to MHSs.

### Differences in MHS use between the education levels

Education level is an important indicator of an individual’s socioeconomic status [12], and is considered one of the predisposing factors toward the use of healthcare services [24]. Many studies have found that those with a higher education level use MHSs more frequently [12,16,18,19,32]. The findings of the present study concur with that finding, in that the subjects with education that extended beyond the high school level were more likely to use such services than those who left the education system before high school. Furthermore, those with higher-level education preferred specialized MHS institutions to primary care centers [19,34]. In addition, one study found that patients with college degrees or higher who suffered from depression were more likely to receive care from a psychiatrist [16]. Thus, the type of MHS institution and service provider (doctor, nurse, or counselor) could vary according to education level. However, this factor could not be considered in the present study since the type of MHS used was unknown. It should be noted that higher education was found to be associated with low prejudice against mental diseases, and particularly depression [14]. Those with a higher level of education generally have a positive attitude toward the effectiveness of psychiatric treatment [15,34], which enhances their use of MHSs; conversely, the economic burden associated with service use is generally higher [15,19] and the level of awareness for psychiatric problems and treatment lower for those with less education, thus hindering MHS use in that group [12]. Therefore, in order to enhance the use of MHSs among relatively uneducated people suffering from depression, an education program that includes information on the detection of depression symptoms and MHS use should be provided to improve their mental health literacy.

### Differences in MHS use between income levels

Income, which like education level is an indicator of socioeconomic status [12], is also a factor that promotes the use of healthcare services [24]. However, the present study found that the use of MHSs did not differ significantly with the monthly family income. Similar results have been reported elsewhere [11,12,35]. Like many European countries, Korea also has a comprehensive health insurance program that covers almost the entire population for mental healthcare. Thus, people with psychiatric diseases and a low income can use MHSs without suffering an excessive financial burden [11]. By contrast, the severity of psychiatric diseases was reported to be higher among those with a low socioeconomic status [11,28], leading to more frequent use of the MHSs [26,27]. As a result, MHS use is higher among the low-income population.

### Limitations

This study was subject to a few limitations. First, the severity and the duration of a depressive mood and the presence of co-morbid mental health issues such as anxiety, which may act as strong confounders regarding the association between sociodemographic characteristics and MHS use, could not be considered. Also, the type of MHS institutions and service providers used were not determined. Second, the use of data from a national health survey may suffer from respondent bias. The use of self-report measures for both depressive mood and MHS use may lead to biases either due to recall or perceived stigma. There would be the discordance in time periods for outpatient use and the measure of a depressive mood. Third, these survey data prevented us from exploring important information on the use of pharmacotherapy such as antidepressants. Therefore, observed differences in the MHS use may not directly reflect differences in the need of MHS use. Fourth, subjects with a depressive mood were not screened using a standardized assessment tool since the data were collected from a general health survey and not a specialized mental health survey. Depressive moods were assessed by a single question in this study; previous studies have investigated the accuracy of such a single-question method, such as a Yale study measuring the accuracy of the following question: “Do you often feel sad or depressed?” The study showed that this question had a sensitivity of 86%, a specificity of 78%, a positive predictability of 82%, and a negative predictability of 82% in screening for depression in patients with recent stroke [36]. Thus, a single question has the potential to be a rapid and reasonable alternative to more lengthy questionnaires in surveys involving large samples [37].

Despite these limitations, this nationwide representative study provides detailed information on the current status of MHS use among subjects with a depressive mood according to their sociodemographic factors, and identified vulnerable social groups for MHS use in Korea. Furthermore, since the KNHANES is conducted every year, future studies will be able to monitor the trend of MHS use among subjects with a depressive mood.

## Conclusions

The findings of this study suggest that the use of MHSs differs among Korean subjects with a depressive mood according to sociodemographic factors. The elderly, adults with a lower education level, and the employed were less likely to use MHSs. This study shows the relationship between sociodemographic factors and the MHS use in Korea by using a nationwide representative data, despite some strong limitations including recall bias and lack of measuring important confounders. The results in this study may be a useful data for policy makers and mental health professionals in improving the public strategy of the mental health delivery system. In order to enhance the use of MHSs, mental health promotion strategies, including community outreach service, campaigns and education programs, should be targeted according to the characteristics of the population.

## Abbreviations

MHS: Mental health service
KNHANES: Korea National Health and Nutrition Examination Survey
KCDC: Korea Centers for Disease Control and Prevention
SE: Standard error
